# Smoker’s Mustache Revisited: Upper Lip Hair Yellow Discoloration Associated With Tobacco

**DOI:** 10.7759/cureus.18988

**Published:** 2021-10-23

**Authors:** Jennifer Laborada, Philip R Cohen

**Affiliations:** 1 Dermatology, University of California Riverside School of Medicine, Riverside, USA; 2 Dermatology, University of California Davis Medical Center, Sacramento, USA

**Keywords:** yellow, xanthotrichia, tar, tobacco, smoker, nicotine, nail, mustache, hair, cigarette

## Abstract

Yellow hair discoloration (xanthotrichia) has been observed in several settings. Indeed, acquired xanthotrichia, in addition to environmental and occupational causes, can be observed secondary to either iatrogenic, topical, or systemic exposure to systemic drugs and certain systemic conditions: most commonly essential fatty acid deficiencies, protein deficiency, or vitamin B12 deficiency. Smoker’s mustache refers to the acquired yellow discoloration of previously white hair on the cutaneous upper lip of men. These individuals are typically elderly and have a history of smoking either cigarettes, cigars, or pipes of several years’ duration. The asymptomatic dyschromia often originates centrally, affecting the hair overlying the philtrum and expanding laterally. The condition is asymptomatic, and affected individuals are either unaware of the color change or not concerned with their altered appearance. Yellow to brown discoloration of the thumbnails, fingernails, or both (such as nicotine sign and/or harlequin nails) may be an accompanying clinical stigma to the smoker’s mustache and a clue to the diagnosis. Management options include smoking cessation or hair removal of the discolored hair, or both; however, patients usually elect to continue smoking, maintain their facial hair, and continue to display their distinctive yellow smoker’s mustache.

## Introduction

Yellow discoloration of the skin and mucous membranes has been associated with several diseases. Yellow skin can be noted in patients with biliary or hepatic disease, renal failure, endocrine disorders, inflammatory and autoimmune disease, and malignancy. In addition, patients with gallstones, liver disease, and pancreatic cancer often have yellow sclera [1].

Acquired yellow hair is referred to as xanthotrichia. There are several potential causes of yellow hair. It can occur secondary to environmental and occupational causes, iatrogenic etiologies, and systemic conditions [2-5].

Smoker’s mustache describes the yellow discoloration of hair on the cutaneous upper lip. This benign condition is mostly seen in Caucasian men with either light-colored hair or age-associated white hair. Tobacco by-products, such as tar and nicotine, have been postulated to cause yellow discoloration. It is believed that smoking cessation would lead to spontaneous resolution of the hair discoloration; however, most affected men wish to continue smoking. In this article, the features of three men with smoker’s mustaches are described: two of them also had yellow-brown pigmentation on their fingernails, tips of their fingers, or both.

## Case presentation

Case 1: A 54-year-old Asian man with a history of depression presented with a one-month rash on his arms. A cutaneous exam revealed lichenified plaques on hands and distal arms, which were diagnosed as dermatitis. He was concurrently treated systemically with a 12-day course of prednisone and topically with twice daily application of 0.1% triamcinolone ointment. His dermatitis resolved completely.

**Figure 1:**
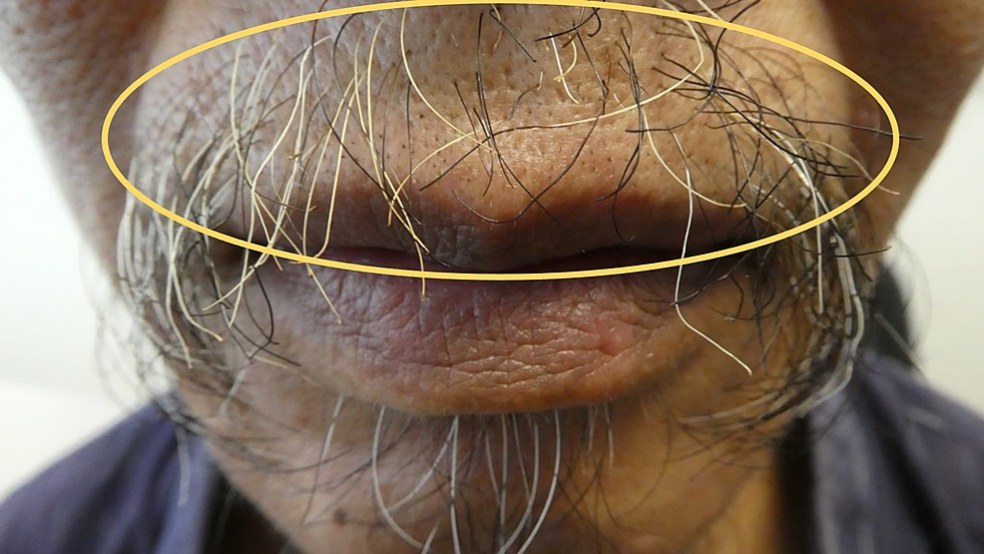
Smoker’s mustache presenting as yellow hair discoloration on the upper lip Acquired yellow hair in the central mustache on the upper lip of a 54-year-old Asian man (yellow oval).

**Figure 2:**
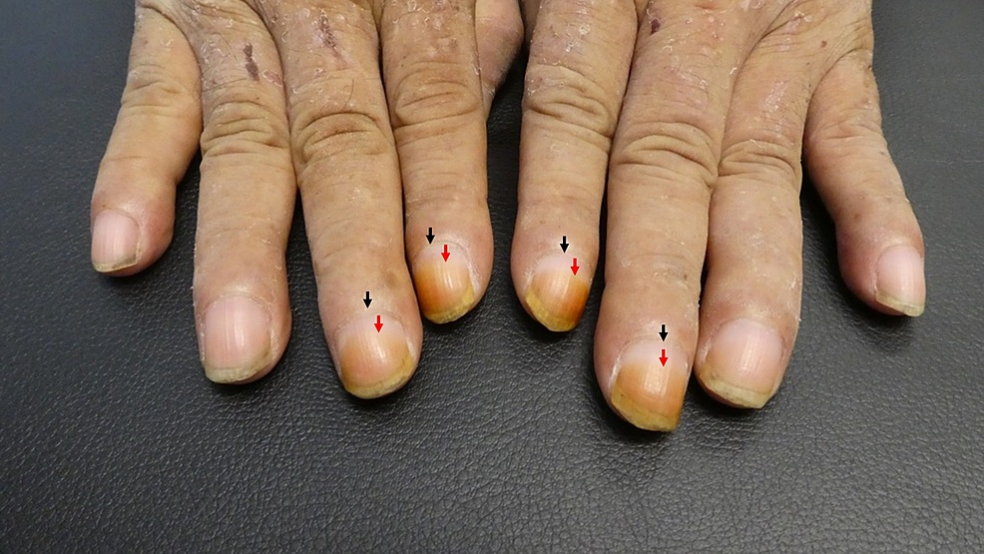
Cigarette smoking-associated harlequin nails There is a sharp line of demarcation between the proximal normal-colored nail (red arrows) and distal brown discoloration of the index and middle fingernails (black arrows). Time of smoking cessation can be calculated assuming a fingernail growth rate of 3 millimeters per month. There is about 1 millimeter of normal nail beneath the proximal nail fold and an additional 3 millimeters of the visible normal-appearing nail; 4 millimeters would correspond to the six weeks he had not been smoking.

Examination of his face showed a sparse mustache, which had yellow discoloration of the hair (Figure 1). Brown discoloration on the distal fingernails on the second and third digits of both hands was also noted (Figure 2). The proximal nail, from the proximal nail fold (also referred to as the cuticle) and extending approximately 3 millimeters distally before the brown nail plate, was normal in appearance. 

This patient uses both hands to hold a cigarette. He has been smoking since his 20s; however, he had stopped smoking six weeks before his appointment. The history of smoking cessation temporally correlates with the newly emerging proximal non-pigmented nail on his second and third digits that distally showed brown discoloration; this physical sign is known as the harlequin nail.

Case 2: A 71-year-old Caucasian man with a history of benign prostatic hyperplasia, cachexia, chronic obstructive pulmonary disease, dementia, gastritis, hypertension, hypothyroidism, unsteady gait, and a 59 pack-year history of cigarette smoking presented for a skin check. Cutaneous examination revealed red scaly plaques on his posterior neck, scalp, and chest. He was diagnosed to have seborrheic dermatitis and was successfully treated with twice daily application of 0.1% triamcinolone cream for five days.

His skin examination also revealed keratotic plaques on his dorsal hands, which were diagnosed as actinic keratosis. The lesions were treated with cryotherapy using liquid nitrogen.

**Figure 3:**
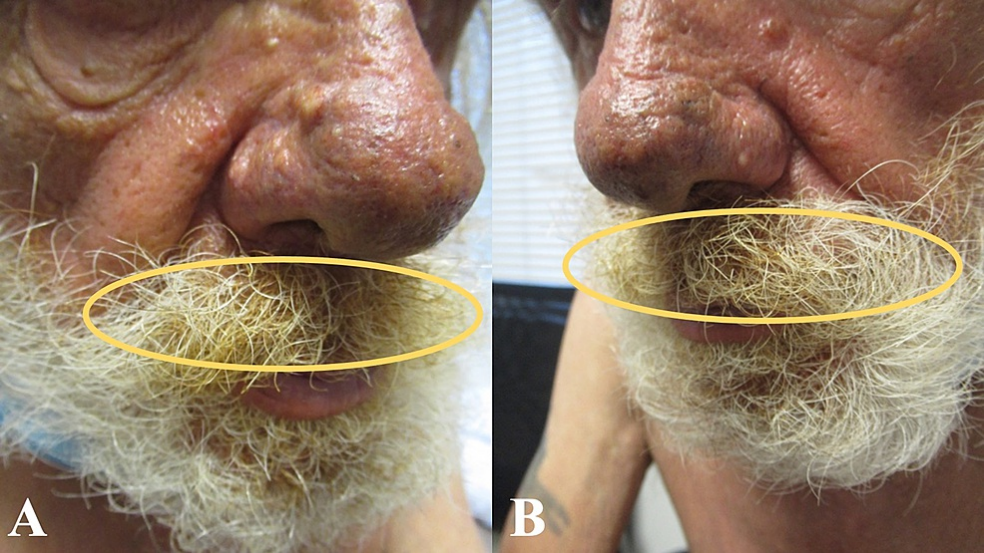
Prominent xanthochromia as the presenting feature of smoker’s mustache Right (A) and left (B) views of prominent yellowing of the mustache (yellow ovals) of a 71-year-old Caucasian man with chronic obstructive pulmonary disease and a 59 pack-year history of cigarette smoking.

**Figure 4:**
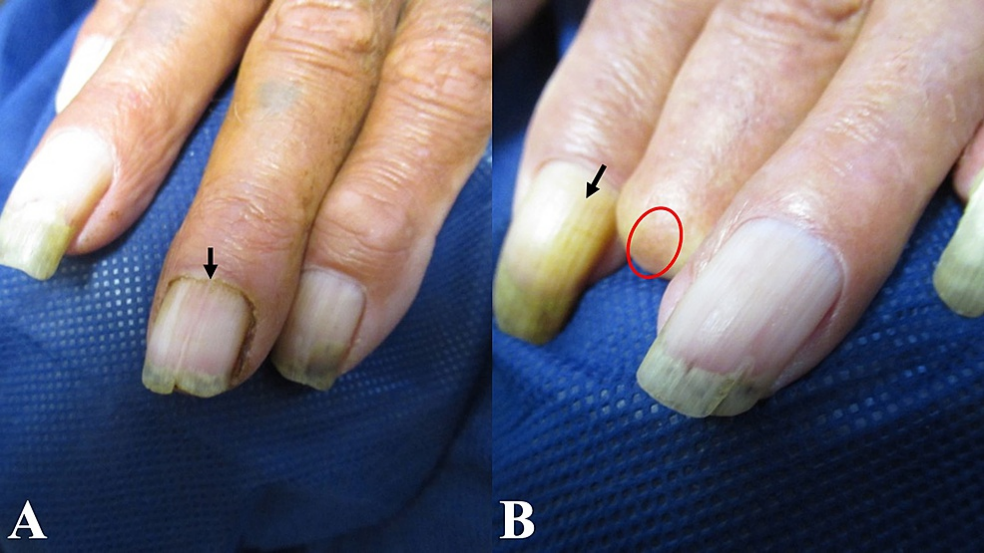
Smoking-associated finger and fingernail discoloration Yellow-brown staining of the right middle fingernail (A) and left index fingernail (B) (black arrows). There is also smoking-related discoloration at the tip of the amputated left middle finger (red oval).

In addition, an examination of his hair and nails was performed. His face showed yellow discoloration of the mustache hair in the central area (Figure 3). Also, there was brown discoloration of the distal third digit, at the distal amputated tip, on his left hand. The nails on the index finger and ring finger of his right hand and the index finger of his left hand also showed brown discoloration (Figure 4).

Case 3: A 71-year-old Caucasian man with a history of cigarette smoking, chronic obstructive pulmonary disease, depression, hypertension, intravenous drug abuse, and type 2 diabetes mellitus presented for a total body skin examination. Diffuse keratotic plaques were noted on the scalp, arms, and legs. A diagnosis of actinic keratosis was established. The lesions were treated with cryotherapy using liquid nitrogen.

**Figure 5:**
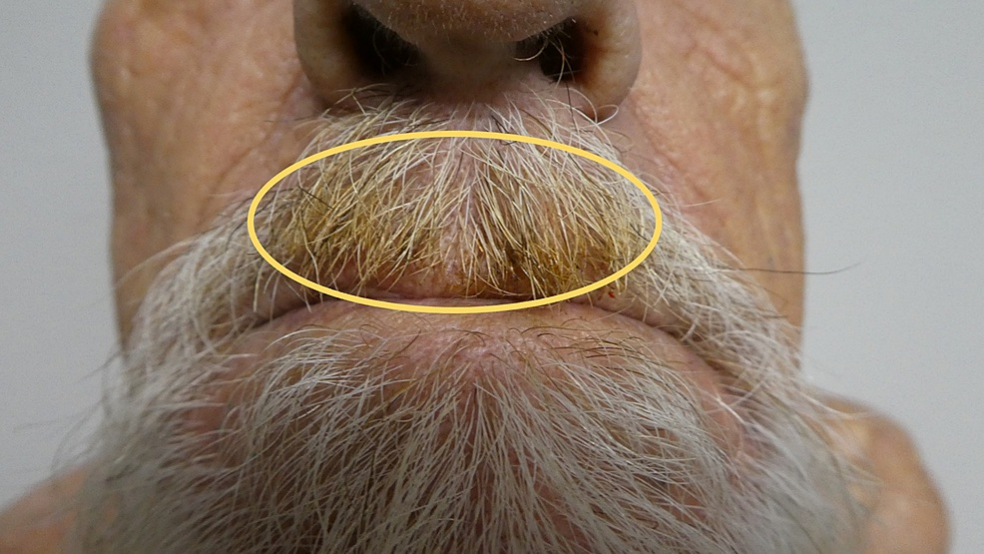
Smoker’s mustache manifested by xanthochromia of the hair on the upper lip of a 71-year-old Caucasian man The yellow discoloration is most noticeable on the central mustache hairs (yellow oval).

The cutaneous examination also showed a yellow discoloration of his central upper mustache hair (Figure 5). There was no discoloration of his fingers or fingernails. He smoked at least four cigarettes per day for more than 50 years.

## Discussion

Dimmick and Wagner introduced the term “smoker’s mustache” in 2006 (Table 1) [3,4]. Subsequently, a case series of five men were added to the literature by Beutler and Cohen in 2015 [4]. The salient features of three additional men with this phenomenon are summarized in this paper.

**Table 1:** Previous reports of smoker’s mustache CR: current report

Authors	Year of publication	Comment	Reference
Dimmick and Wagner	2006	Case report of a white-haired, 55-year-old Caucasian man who had smoked three-fourths of a pack of cigarettes daily for the past 35 years. The midline region of his white mustache displayed a yellowish pigmentation of the distal hairs with sparing of the proximal portions. His cutaneous examination was consistent with his habit of always holding his cigarette directly below the central region of his mustache.	[3]
Beutler and Cohen	2015	Largest case series of five Caucasian men with tobacco-associated yellow discoloration of their mustache. Three of the five men also displayed brown discoloration of their distal fingers and/or fingernails.	[4]
Laborada and Cohen	2021	Case series of three patients (two Caucasian and one Asian) with a smoker’s mustache. Two of the men also had brown discoloration of their fingernails and/or finger.	[CR]

Smoker’s mustache has been reported, including the men in this paper, in nine individuals. Eight of the men were Caucasian, and one man was Asian. The age at diagnosis ranged from 54 to 71 years old (median age, 63 years); however, the yellow discoloration may likely have been present for several years before being noticed during the patient’s evaluation by his dermatologist.

Xanthotrichia of the mustache was always asymptomatic. Indeed, it was frequently an incidental finding. Most of the patients were either unaware of its presence or unconcerned with the color change.

Associated features in some of the men with a smoker’s mustache included discoloration of the fingertips, fingernails, or both. Indeed, in the previously published case series, three of five (80%) of the men had yellow-brown discoloration at these locations [4]. In this report, two patients had discoloration of the fingernails and/or fingertips.

Nicotine sign refers to the yellow discoloration resulting from continued staining of the nails with tobacco by-products [6]. Harlequin nail is a distinct physical sign that was described in 1990 by Verghese et al. since they considered the affected fingernails to be fancifully varied in color; it presents as a sharp line of demarcation between the distal tobacco by-products-stained nail and proximal growth of the normal nail [7]. Fingernails grow approximately 3 millimeters per month. The harlequin nail enables the clinician to calculate when a patient has abruptly stopped smoking, often due to a severe illness such as a stroke [6-8]. Our patient, a 54-year-old Asian man, had approximately 3 millimeters of normal-appearing nail distal to the proximal nail fold; assuming at least 1 millimeter of normal nail beneath his proximal nail fold, this would total 4 millimeters and corresponds to his smoking cessation about one and a half months earlier. 

There are limited, albeit effective, treatments for yellow discoloration of the upper lip hair secondary to smoking. These include either cessation of smoking, shaving the mustache, or both. However, most of the men do not consider these to be favorable options and elect to maintain their mustache xanthotrichia.

Scalp hair can also become yellow, secondary to exposure to tobacco by-products. Accidental staining of the hair by tobacco tars can result in yellow or yellow-brown discoloration of white or grey hair. Often, patients are not aware of the cause of their hair discoloration [2].

Indeed, Kellen et al. described an interesting neuro-ophthalmological sign. They observed a 63-year-old Caucasian man who had a significant smoking history and presented with painless vision loss, accompanied by a yellow forelock of scalp hair, of two months' duration. Hair sample analysis revealed a higher nicotine content at the forelock compared to the occiput, which ultimately served as a clinical clue to the diagnosis of tobacco-related amblyopia [9].

In addition to tobacco by-products, there are several causes of acquired xanthotrichia. Some of these include environmental and occupational etiologies. Exogenous agents that have been observed to cause yellow hair discoloration include dihydroxyacetone, hydrogen peroxide, hypochlorous acid, picric acid, resorcinol, sunlight exposure, 1-naphthol derivatives, and 4,4’-methylenedianiline [2-5]. Except for hydrogen peroxide, these etiologies do not cause hair loss; however, all of them can be associated with cutaneous and systemic adverse events (Table 2) [10-17].

**Table 2:** Summary of adverse effects of environment and occupational etiologies associated with xanthochromia YHD: yellow hair discoloration; HL: hair loss; Ref: reference; +: positive; -: negative; GI:, gastrointestinal

Etiology	YHD	HL	Cutaneous adverse effects	Systemic adverse effects	Ref
Dihydroxyacetone	+	-	Allergic contact dermatitis (rare: two per 100,000 people)	DNA damage	[10]
Hydrogen peroxide	+	+	Chemical burns, dermatitis, scalp burns	High concentration can cause poisoning as a result of corrosive damage, direct cytotoxicity from lipid peroxidation, and oxygen gas formation (with embolism and central nervous system damage)	[11]
Hypochlorous acid	+	-	Chemical burns, dermal hypersensitivity, eye irritation, immediate or delayed-type skin reactions	Corrosive GI injury, hypernatremia, hyperchloremia, metabolic acidosis	[12]
Picric acid	+	-	Allergic contact dermatitis, skin sensitizer	Diarrhea, dizziness, headache, nausea, vomiting	[13]
Resorcinol	+	-	Irritant contact dermatitis	Bradycardia, diarrhea, drowsiness, headache, nausea, nervousness, restlessness, shortness of breath, stomach pain, tiredness, vomiting, weakness	[14]
Sunlight exposure	+	-	Melanoma, non-melanoma skin cancer, photosensitivity, premature aging, sunburn	Cataracts, immune system suppression	[15]
1-naphthol derivatives	+	-	Irritant contact dermatitis	Cataracts, DNA damage, hemolytic anemia, laryngeal tumors, liver damage, neurologic damage	[16]
4,4’ methylenedianiline	+	-	Allergic contact dermatitis	Hepatotoxin	[17]

Drug-associated yellow hair discoloration has also been observed. It can result from exposure to various systemic medications. These include chloroquine, cisplatin, heptaminol, mephenesin, minoxidil, para-aminobenzoic acid, sunitinib, tamsulosin, and valproic acid [2-5].

Other causes for medication-related yellow hair are topical agents. Application of anthralin, minoxidil, selenium sulfide, and tar shampoo has resulted in xanthotrichia [2-5]. Recently, a 77-year-old woman repeatedly developed yellow hair following sequential application of bacitracin zinc ointment and selenium sulfide 2.5% lotion; spontaneous resolution of her scalp hair xanthochromia occurred when she stopped applying these products to her scalp [5].

Systemic conditions can also be associated with the acquisition of yellow hair. Most of the disorders are related to diminished dietary intake. The conditions include essential fatty acid deficiency, protein-calorie malnutrition, and vitamin B12 deficiency [2-5].

## Conclusions

Xanthotrichia, acquired yellow hair discoloration, can result from environmental and occupational exposures, iatrogenic etiologies, topical agents, systemic drugs, or systemic conditions. Smoker’s mustache is a unique presentation of yellow discoloration of previously white hair on the cutaneous upper lip secondary to tobacco by-products such as nicotine and tar. It is asymptomatic and often an incidental finding. Smoker’s mustache typically affects hair centrally located at the philtrum and may subsequently expand to involve laterally located hair on the upper lip. Examination of the fingertips and fingernails may also demonstrate tobacco-associated yellow to brown discoloration: nicotine sign or harlequin nails. Treatment options for men with a smoker’s mustache include smoking cessation, hair removal by shaving or using other modalities, or both; however, most patients elect to continue smoking and maintain the distinctive yellow dyschromia of their mustache.
